# Relationship between postoperative biomarkers of neuronal injury and postoperative cognitive dysfunction: A meta-analysis

**DOI:** 10.1371/journal.pone.0284728

**Published:** 2023-04-25

**Authors:** Xiaohua Wang, Xinli Chen, Fan Wu, Yingchao Liu, Yushen Yang, Weican Chen, Zhigang Pan, Weipeng Hu, Feng Zheng, Hefan He

**Affiliations:** 1 Department of Anesthesiology, The Second Affiliated Hospital of Fujian Medical University, Quanzhou, Fujian Province, China; 2 Department of Neurosurgery, The Second Affiliated Hospital of Fujian Medical University, Quanzhou, Fujian Province, China; National Institute of Mental Health and Neuro Sciences, INDIA

## Abstract

Early biomarkers are needed to identify patients at risk of developing postoperative cognitive dysfunction (POCD). Our objective was to determine neuronal injury-related biomarkers with predictive values for this condition. Six biomarkers (S100β, neuron-specific enolase [NSE], amyloid beta [Aβ], tau, neurofilament light chain, and glial fibrillary acidic protein) were evaluated. According to the first postoperative sampling time, observational studies showed that S100β was significantly higher in patients with POCD than in those without POCD (standardized mean difference [SMD]: 6.92, 95% confidence interval [CI]: 4.44−9.41). The randomized controlled trial (RCT) showed that S100β (SMD: 37.31, 95% CI: 30.97−43.64) and NSE (SMD: 3.50, 95% CI: 2.71−4.28) in the POCD group were significantly higher than in the non-POCD group. The pooled data of observational studies by postoperative sampling time showed significantly higher levels of the following biomarkers in the POCD groups than in the control groups: S100β levels at 1 hour (SMD: 1.35, 95% CI: 0.07−2.64), 2 days (SMD: 27.97, 95% CI: 25.01−30.94), and 9 days (SMD: 6.41, 95% CI: 5.64−7.19); NSE levels at 1 hour (SMD: 0.92, 95% CI: 0.25−1.60), 6 hours (SMD: 0.79, 95% CI: 0.12−1.45), and 24 hours (SMD: 0.84, 95% CI: 0.38−1.29); and Aβ levels at 24 hours (SMD: 2.30, 95% CI: 1.54−3.06), 2 days (SMD: 2.30, 95% CI: 1.83−2.78), and 9 days (SMD: 2.76, 95% CI: 2.25−3.26). The pooled data of the RCT showed that the following biomarkers were significantly higher in POCD patients than in non-POCD patients: S100β levels at 2 days (SMD: 37.31, 95% CI: 30.97−43.64) and 9 days (SMD: 126.37, 95% CI: 104.97−147.76) and NSE levels at 2 days (SMD: 3.50, 95% CI: 2.71−4.28) and 9 days (SMD: 8.53, 95% CI: 7.00−10.06). High postoperative levels of S100β, NSE, and Aβ may predict POCD. The relationship between these biomarkers and POCD may be affected by sampling time.

## Introduction

Postoperative cognitive dysfunction (POCD) affects direction, attention, consciousness, perception, and judgment of patients under general anesthesia [1], with an incidence of 8.9–46.1% [2]. Several factors, including neuroinflammation, oxidative stress, autophagy disorder, neuronal injury, and lack of neurotrophic support are hypothesized to contribute to POCD [3, 4]. However, the specific pathogenesis of POCD remains unclear. Currently, the diagnosis of POCD is based mainly on neuropsychological tests. However, the composition of the test batteries varies greatly [5] and they are easily affected by factors such as culture, educational level, and language [6], which leads to their limited value for diagnostic application. Recent studies have found that anesthesia and surgery can cause neuronal damage and related biomarkers may be associated with postoperative cognitive outcomes [7, 8]. Identifying the relationship between neuronal injury biomarkers and POCD may present another method of diagnosing POCD.

Several biomarkers have been confirmed to be associated with neurodegenerative diseases. Amyloid beta (Aβ) and tau proteins are included in the diagnostic guidelines for Alzheimer’s disease (AD) [9]. Neurofilament light (NFL) is associated with the pathophysiology of amyotrophic lateral sclerosis, Guillain-Barré syndrome, Parkinson’s disease, and AD [10–13]. S100β, neuron-specific enolase (NSE), and glial fibrillary acidic protein (GFAP) have been reported to be associated with the pathophysiology of stroke and traumatic brain injury [14–18]. However, the relationship between these biomarkers and POCD remains unclear. Therefore, we performed a meta-analysis of the relationship between neuronal damage biomarkers and POCD to explore whether these biomarkers have predictive value for this condition. The results of our analysis showed that the three biomarkers, S100β, NSE, and Aβ, have predictive value for POCD.

## Materials and methods

### Sources and search strategy

We conducted a systematic literature review using the PubMed, Embase, and Cochrane databases to identify studies published up to April 2022. To bring together meaningful results, we focused more on neuronal damage markers that have been extensively studied [15, 19–22]. The search terms included the following keywords and their combinations: (“S100β” OR “neuron-specific enolase” OR “amyloid beta” OR “tau” OR “neurofilament light” OR “glial fibrillary acidic protein” OR “neuronal injury”) AND (“postoperative cognitive dysfunction”).

### Study selection

To be included, studies had to meet the following criteria: (1) conducted in humans; (2) assessment of at least one specific neuronal marker in blood or cerebrospinal fluid (CSF); and (3) written in English. Studies with the following characteristics were excluded: (1) no clear differentiation between POCD subtypes, (2) lack of comparison groups, (3) non-human studies, and (4) non-English articles. Two reviewers (X.W. and X.C.) independently evaluated the retrieved articles according to the title, summary, or full text, and a third independent reviewer (W.C.) resolved discrepancies.

### Data extraction and synthesis

Data extraction, synthesis, and a risk of bias analysis were guided by Preferred Reporting Items for Systematic Reviews and Meta-Analysis (S1 Appendix). Using standardized extraction tables, we recorded the following data: (1) authors and publication year, (2) research design, (3) sample size and participant characteristics, (4) type of surgery, (5) type of anesthesia, (6) biomarkers and sampling time and source, and (7) methods to diagnose POCD. For studies reporting only the median, range, or interquartile range, we applied the appropriate formulas to convert them to mean and standard deviation [23–25]. The data used for all analyses can be seen in S2 Appendix.

### Assessment of study quality

Risk of bias was assessed for the individual randomized controlled trial (RCT) according to the Cochrane Collaboration Tool, which evaluated trials based on the presence or absence of randomization sequence generation, allocation concealment, selective reporting, blinding of participants and personnel, blinding of outcome assessment, incomplete outcome data, and other forms of bias [26]. The quality of observational articles was independently classified using the Newcastle–Ottawa Scale [27]. The Newcastle–Ottawa Scale score ranges from 0 to 9 stars. A quality score was calculated based on three major components: (1) selection of study groups (0–4 stars), (2) comparability of study groups (0–2 stars), and (3) determination of the exposure and outcome of interest in case-control and cohort studies (0–3 stars). Studies with scores of 7 to 9, 4 to 6, and 0 to 3 stars were considered high, medium, and low quality, respectively. Disagreements were resolved by discussion and consensus.

### Statistical analysis

If two or more studies reported factors of interest, the standardized mean difference (SMD) and the 95% confidence interval (CI) of each result were calculated using the random-effects model to evaluate the association between markers and POCD. The chi-square test was used to calculate the Q-value to quantify the heterogeneity between joint tests, and the I^2^ index was used to determine the impact of heterogeneity in inconsistent calculations. The significance level of the heterogeneity *P*-value was established at 0.1, and the I^2^ statistic was interpreted as follows: 0–40%, low; 30–60%, moderate; 50–90%, substantial; and 75–100%, considerable heterogeneity. Statistically significant heterogeneity was considered present at *P* <0.1 and I^2^ >50%. Because the sampling time of each study varied greatly, pooled analyses were performed for different time points with more than two studies. Sensitivity analysis was used to test the impact of each study on the overall estimation. The funnel plot asymmetry test was recommended only when at least 10 studies were included in the meta-analysis. Therefore, we used Egger’s test to assess asymmetry and publication bias. Statistical significance was established at *P* <0.05. The meta-analysis was carried out using Review Manager 5.4.1 (Cochrane Org., London, UK), and publication bias was evaluated using Stata 16 (StataCorp LLC., College Station, TX, USA).

## Results

### Study selection

**Fig 1:**
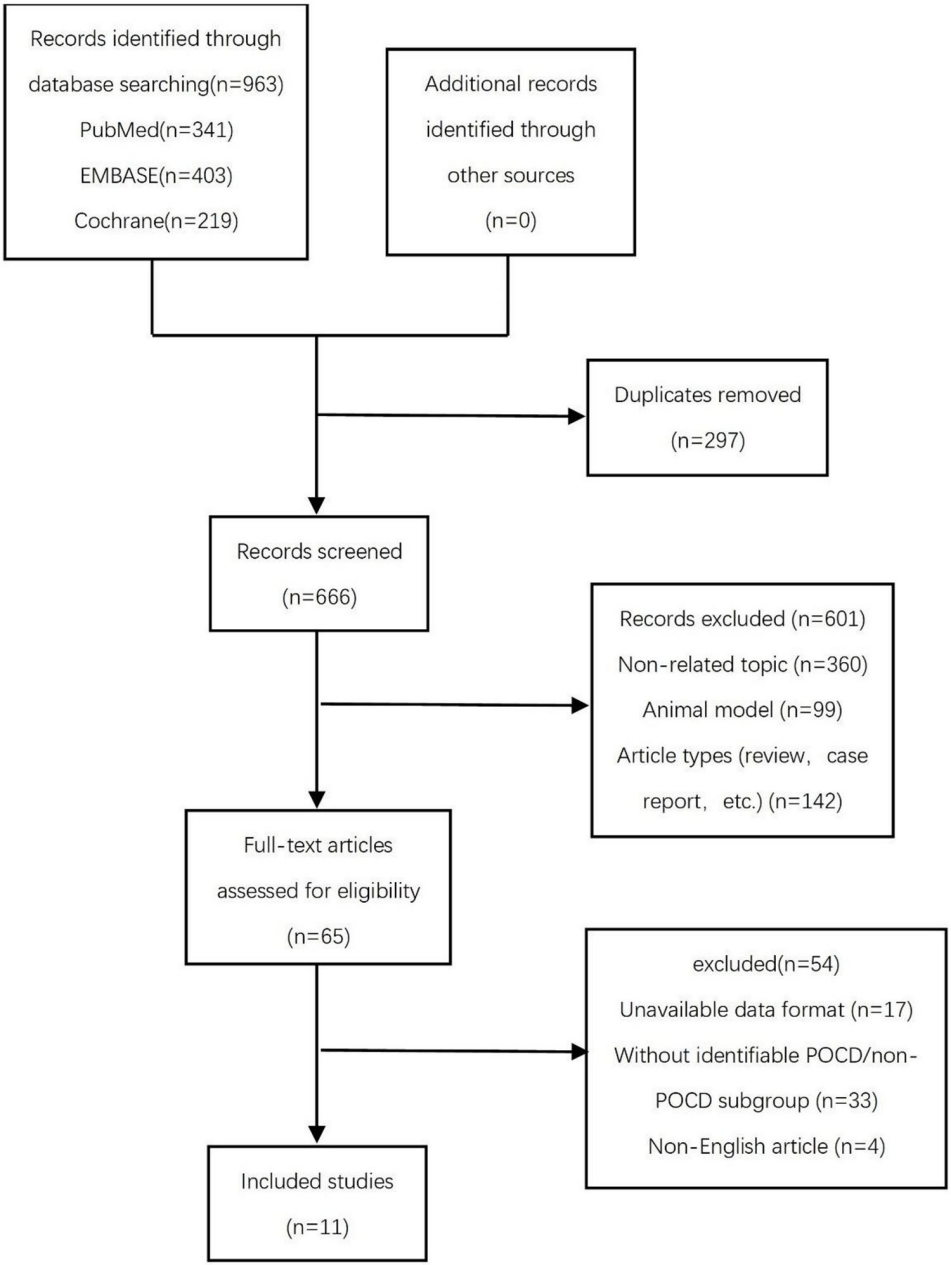
Flow diagram for literature selection.

We found a total of 963 potentially eligible articles, 666 of which were obtained after removing duplicate articles. Subsequently, 601 non-pertinent articles were excluded by evaluating titles and abstracts. In the remaining 65 articles, the full texts were accessed. Ultimately, 11 articles [28–38] were included in the present analysis, including a total of 878 patients where the predictive values of postoperative neuronal injury biomarkers were compared in the POCD and non-POCD groups (Fig 1).

### Study characteristics

The demographic and clinical characteristics of each study are presented in Table 1. The sample sizes of the studies varied greatly (POCD groups, 22.36 ± 11.22; non-POCD groups, 57.45 ± 42.79). The median Newcastle–Ottawa Scale score was 7.6 (S3 Appendix), indicating that the quality of these articles was high. The bias risk assessment suggested that there was little evidence of significant bias in the included RCT (S4 Appendix). The number of studies on tau (n = 1) and NFL (n = 1) was insufficient for meta-analysis, and a descriptive summary of the results was performed. GFAP studies were not included because the data were not available. Therefore, three biomarkers, S100β, NSE, and Aβ, were quantitatively analyzed in the present study.

**Table 1:** Characteristics of the included studies.

Author	Study design	No. of Patients	Age	Nature of Surgery	Patient Population	Anesthesia	Biomarker	Time of Post-op biomarker	Source	POCD Diagnosis
Ying-Hao Cao 2017 [33]	ROS	60	NA	liver transplantation	elective	GA	S100β, NSE	24 hours	Blood	MMSE, DST, Digit Symbol Test, TMT, Short Story Memory Test
PENG YU 2016 [32]	POS	168	64.5±10.7	subtotal gastrectomy	elective	GA	Aβ	2, 9 days	Blood	MMSE, MoCA
Y.-L. CHI 2017 [34]	RCT	142	64.5±8.7	TURP	elective	GA	S100β, NSE	2, 9 days	Blood	MMSE, MoCA
Y-C. Li 2012 [29]	POS	42	>60y	total hip-replacement	elective	GA	S100β	1, 6 hours	Blood	DSST of the Wechsler Adult Intelligence Scale, CET, NCT
Xi-ming LI 2014 [30]	POS	50	40–80	laparoscopic pancreaticoduodenectomy	elective	GA	Aβ	24 hours	Blood	MMSE, DSST, TMT, VFT, Word Recognition Memory Test
X. HE 2017 [35]	POS	178	54.5±12.7	single valve replacement	elective	GA	S100β	2, 9 days	Blood	MMSE, MoCA
Zilin Wan 2021 [38]	POS	40	51±11	total arch replacement	elective	GA	S100β, NSE	1, 6, 24 hours	Blood	MMSE
YOSHUA BAKTIAR 2020 [37]	POS	60	NA	open-heart	elective	GA	S100β	6 hours	Blood	RAVLT, TMT, DST
U. LINSTEDT 2002 [28]	ROS	120	64(18–85)	Multiple (noncardiac, nonhip fracture)	elective	GA	S100β, NSE	30 minutes, 4, 18, 36 hours	Blood	DSST of the Wechsler Adult Intelligence Scale, CET, NCT
Lisbeth Evered 2016 [31]	POS	59	>60y	total hip replacement	elective	combined SA and GA	Aβ, T-Tau, P-Tau, NFL	7 days, 3 months	CSF	CERAD AVLT, TMT, DSST, COWAT, CERAD Semantic Fluency test, GPT
W. F. Kok 2017 [36]	POS	57	NA	coronary artery bypass	elective	GA	NSE, S100β	6, 24 hours	Blood	Detection Task, Identification Task, OCLT, One Back Task

ROS: Retrospective observational study, POS: Prospective observational study, RCT: Randomized controlled trial, NA: Unavailable, TURP: Transurethral resection of the prostate, GA: General anesthesia, SA: Spinal anesthesia, NSE: Neuron-specific enolase, Aβ: Amyloid beta, NFL: Neurofilament light, CSF: Cerebrospinal fluid, MMSE: Mini-Mental State Examination, DST: Digit Span Test, TMT: Trail Making Test, MoCA: Montreal Cognitive Assessment, DSST: Digit Symbol Substitution Test, CET: Concentration Endurance Test, NCT: Number Connection Test, VFT: Verbal Fluency Test, RAVLT: Rey Auditory Verbal Learning Test, CERAD: Consortium to Establish a Registry in Alzheimer’s Disease, AVLT: Auditory Verbal Learning Test, COWAT: Controlled Oral Word Association Test, GPT: Grooved Pegboard Test, OCLT: One Card Learning Task.

### Results of individual studies

#### Tau and NFL biomarkers

In our included studies, only Evered et al. [31] measured total tau, phosphorylated tau, and NFL biomarkers in the CSF. Because the number of studies was insufficient, a quantitative analysis could not be performed. Qualitative analysis showed that there were no statistically significant differences in these three biomarkers between the POCD and non-POCD groups 7 days or 3 months postoperatively.

#### S100β, NSE, and Aβ biomarkers

**Fig 2:**
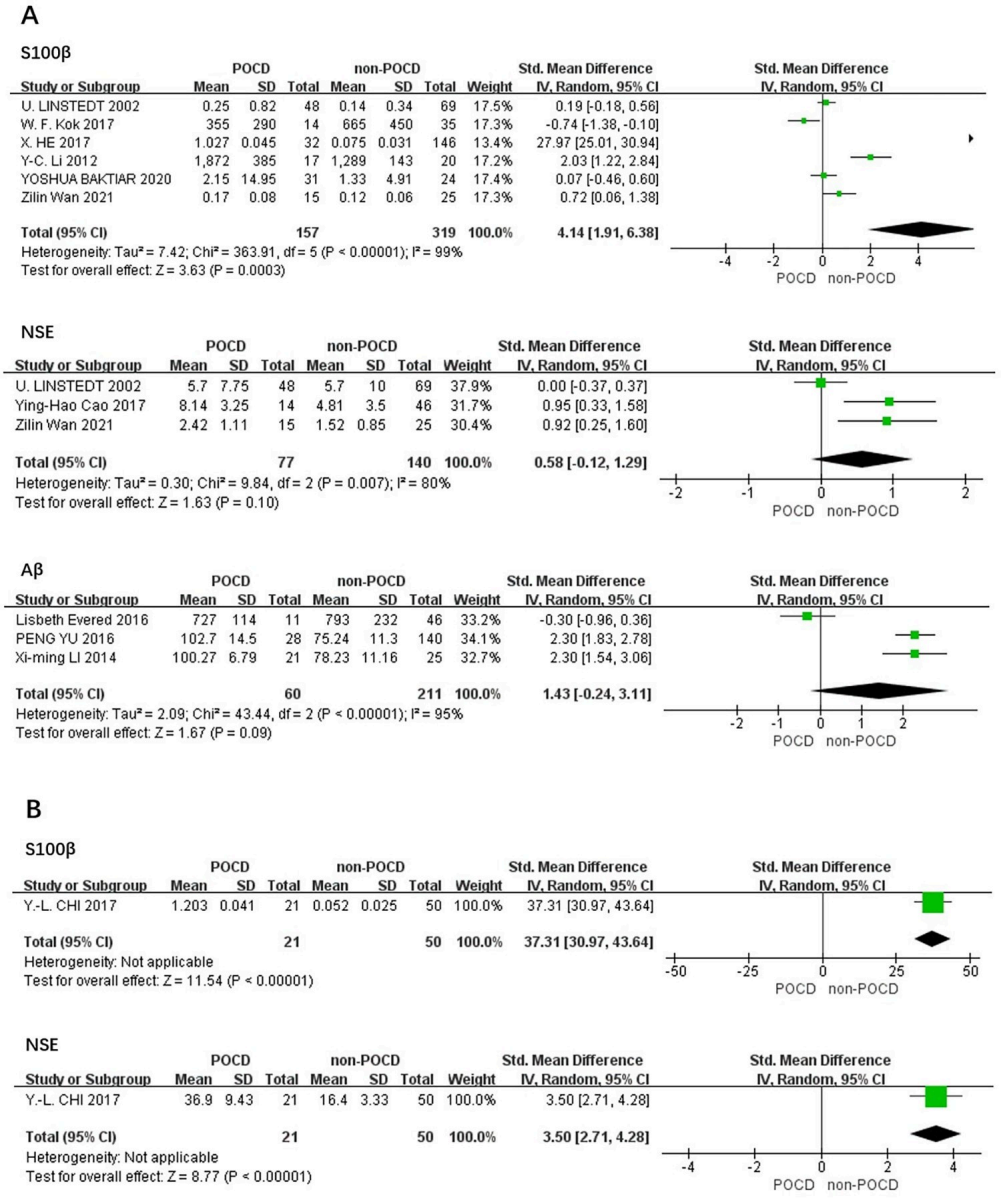
Forest plot of the correlation between neuronal injury biomarkers and POCD by the earliest time of postoperative sampling. (A) shows the results of the observational studies and (B) shows the results of randomized controlled trial.

According to the first postoperative sampling time-point, the observational studies included showed that S100β was significantly higher in patients with POCD than in those without POCD (SMD: 4.14, 95% CI: 1.91−6.38). No significant differences in NSE (SMD: 0.58, 95% CI: -0.12−1.29) and Aβ (SMD: 1.43, 95% CI: -0.24−3.11) were detected between the two groups (Fig 2A). The included RCT showed that S100β (SMD: 37.31, 95% CI: 30.97−43.64) and NSE (SMD: 3.50, 95% CI: 2.71−4.28) were significantly higher in POCD patients than in non-POCD patients (Fig 2B).

**Fig 3:**
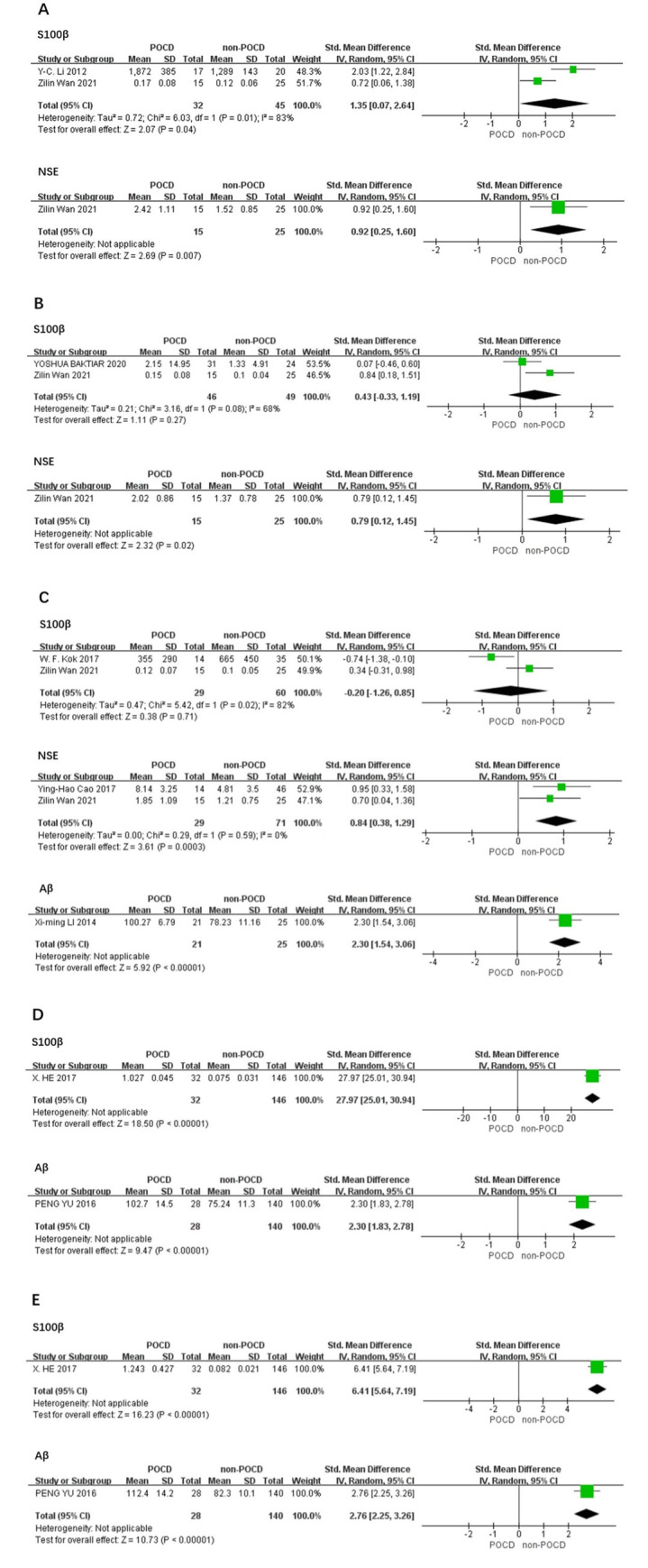
Forest plot of observational studies on the association between neuronal injury biomarkers and POCD. The plots show the correlation at 1 hour (A), 6 hours (B), 24 hours (C), 2 days (D), and 9 days (E) postoperatively.

The pooled data of the included observational studies, according to postoperative sampling time, showed that NSE levels at 1 hour (SMD: 0.92, 95% CI: 0.25−1.60) (Fig 3A), 6 hours (SMD: 0.79, 95% CI: 0.12−1.45) (Fig 3B), and 24 hours (SMD: 0.84, 95% CI: 0.38−1.29) (Fig 3C) postoperatively were significantly higher in the POCD group than in the non-POCD group. Aβ in the POCD group was significantly higher than in the non-POCD group at 24 hours (SMD: 2.30, 95% CI: 1.54−3.06) (Fig 3C), 2 days (SMD: 2.30, 95% CI: 1.83−2.78) (Fig 3D), and 9 days (SMD: 2.76, 95% CI: 2.25−3.26) (Fig 3E) postoperatively. S100β levels were significantly higher in POCD patients than in non-POCD patients at 1 hour (SMD: 1.35, 95% CI: 0.07−2.64) (Fig 3A), 2 days (SMD: 27.97, 95% CI: 25.01−30.94) (Fig 3D), and 9 days (SMD: 6.41, 95% CI: 5.64−7.19) (Fig 3E) postoperatively. However, there were no statistically significant differences between the two groups at 6 hours (SMD: 0.43, 95% CI: -0.33−1.19) (Fig 3B) and 24 hours (SMD: -0.20, 95% CI: -1.26−0.85) (Fig 3C) postoperatively.

**Fig 4:**
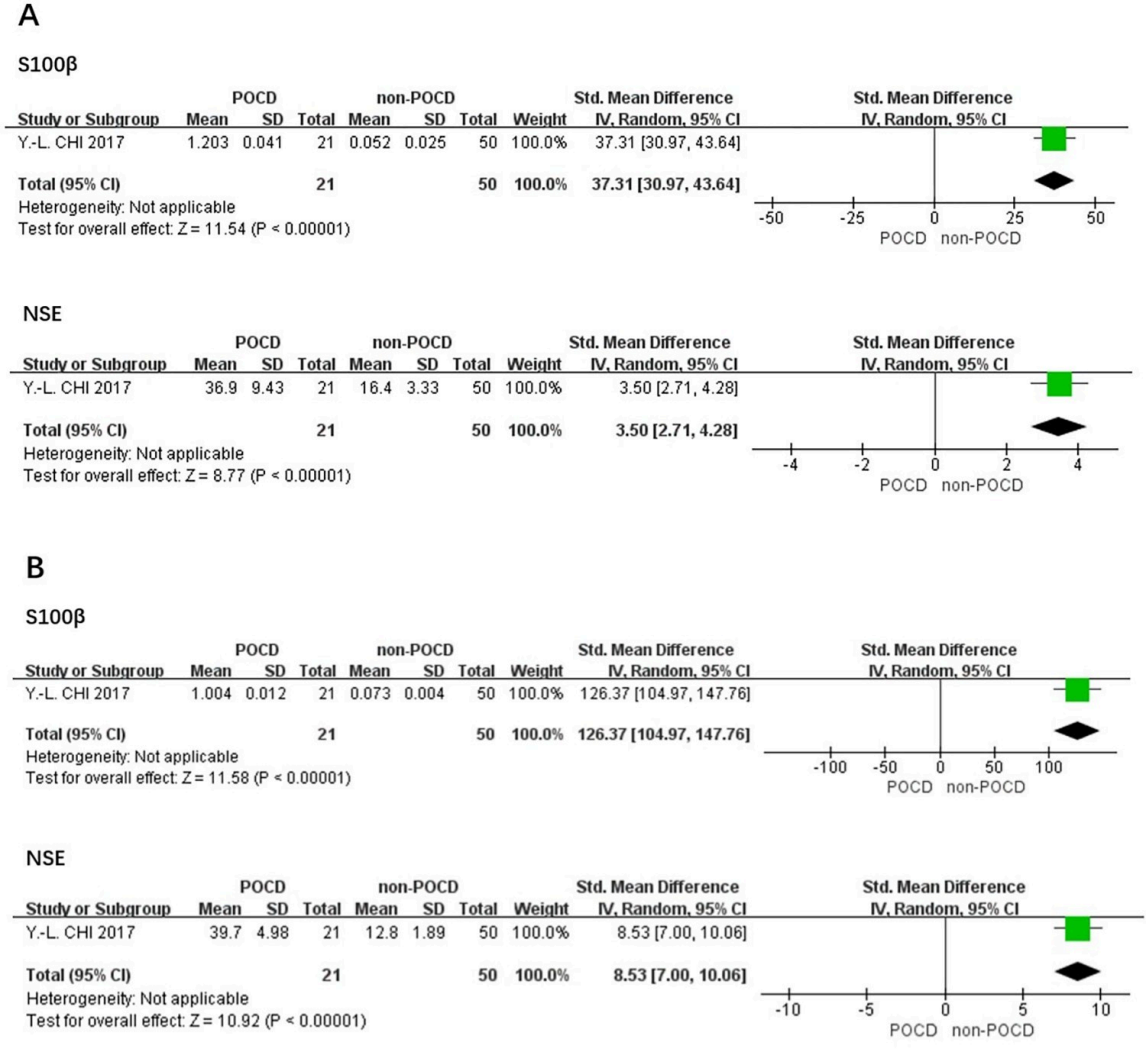
Forest plot of the randomized controlled trial on the association between neuronal injury biomarkers and POCD. The plots show the correlation at 2 days (A) and 9 days (B) postoperatively.

The pooled data of the included RCT showed that S100β levels at 2 days (SMD: 37.31, 95% CI: 30.97−43.64) (Fig 4A) and 9 days (SMD: 126.37, 95% CI: 104.97−147.76) (Fig 4B) postoperatively were significantly higher in the POCD group than in the non-POCD group. NSE in the POCD group was significantly higher than in the non-POCD group at 2 days (SMD: 3.50, 95% CI: 2.71−4.28) (Fig 4A) and 9 days (SMD: 8.53, 95% CI: 7.00−10.06) (Fig 4B) postoperatively.

### Sensitivity analysis

Sensitivity analysis was used to explore the impact of each study on the overall estimation. The results of the sensitivity analysis showed that when the study by He et al. [35] was removed, there was no significant difference in S100β level between POCD patients and non-POCD patients (*P* = 0.25). When the study by Linstedt et al. [28] was removed, the NSE was significantly higher in patients with POCD than in those without POCD (*P* <0.0001). When the study by Evered et al. [31] was removed, Aβ levels were significantly higher in patients with POCD than in those without POCD (*P* <0.00001). This suggests that the results summarized at the first postoperative sampling should be interpreted with caution.

### Publication bias

The Egger test did not show evidence of publication bias (S5 Appendix).

## Discussion

Ntalouka et al. [39] showed that orthopedic and cardiac surgeries were associated with a higher incidence of POCD. In this meta-analysis, cardiac surgery [35–38] and hip replacement surgery [29, 31] represented more than half of the total included studies. The incidence of POCD in the included studies ranged from 16.66% to 56.36%, with the highest incidence being open-heart surgery [37], which may further strengthen the association between biomarkers and POCD.

**Fig 5:**
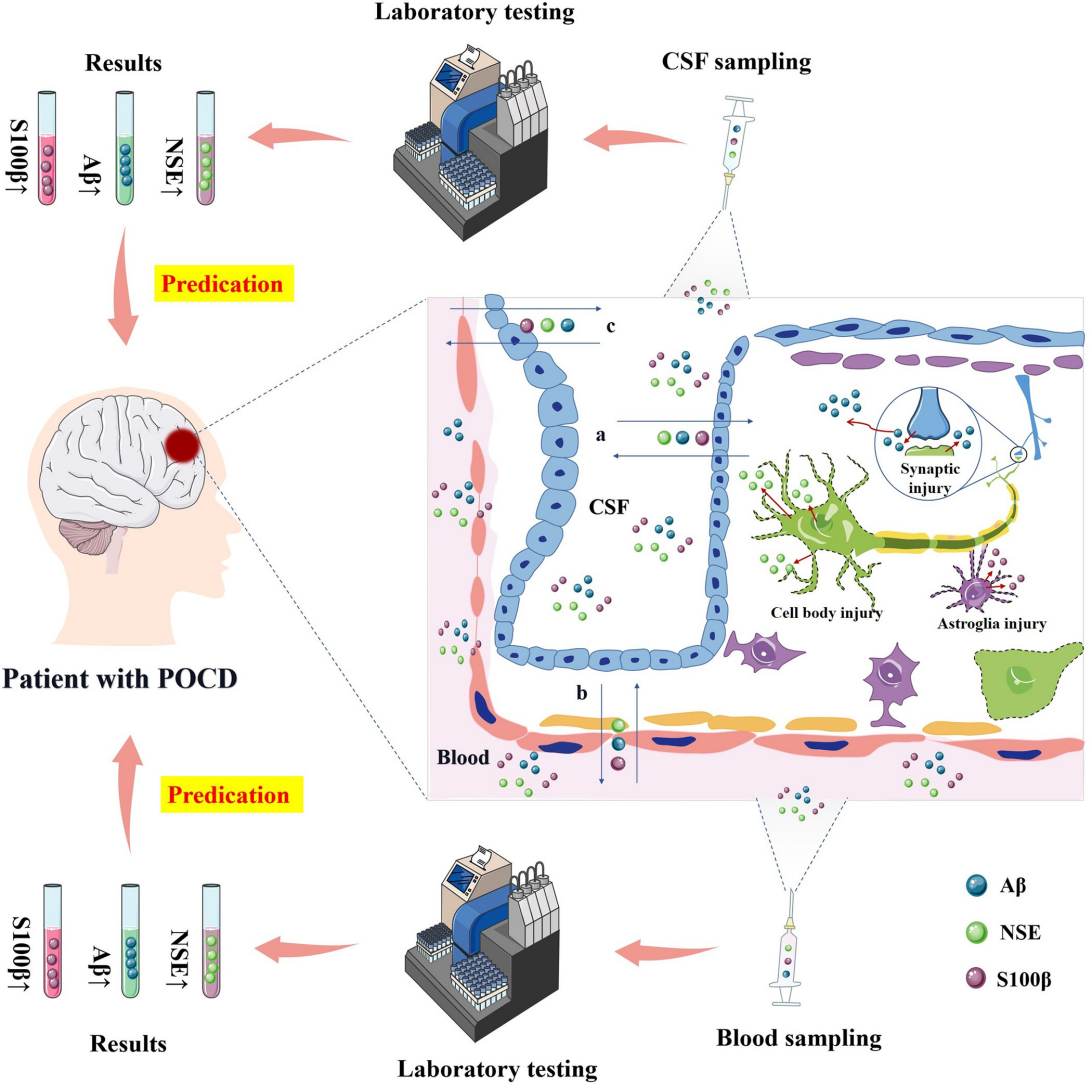
Relationship between neuronal injury and postoperative biomarkers. (A) Brain damage occurs under the stimulation of surgery and anesthesia, injured neurons release the corresponding biomarkers into the CSF and blood through CSF-brain barrier, (B) blood-brain barrier, and (C) blood-CSF barrier. The increase of these biomarkers suggests the occurrence of POCD. NSE: neuron-specific enolase, Aβ: Amyloid beta, POCD: postoperative cognitive dysfunction, CSF: cerebrospinal fluid.

S100β is a 21 kDa protein with a homologous dimer structure that belongs to the calcium-mediated protein family [40]. The protein may be involved in the growth, proliferation, and activation of neurons and glial cells and can be expressed and secreted in the CSF through astrocytes after a central nervous system injury [14]. The present analysis showed that S100β was associated with POCD (Fig 5), which is consistent with a previous finding [41]. However, some studies suggested that S100β was not related to postoperative cognitive results [8, 42, 43]. The pooled data of the included observational studies showed that S100β at 1 hour, 2 days, and 9 days postoperatively was associated with POCD, while S100β at 6 hours and 24 hours postoperatively was not significantly correlated with POCD. Further studies are warranted to confirm this finding. Consistent with the results of the observational studies, the RCT showed that S100β was associated with POCD at 2 days and 9 days postoperatively.

NSE is a dimer enzyme in neurons and neuroendocrine cells and has a half-life of 24 hours [14]. NSE has been confirmed to provide quantitative measures of brain injury and improve the diagnosis and outcome evaluation of many brain-related diseases, such as ischemic stroke, intracerebral hemorrhage, seizures, and traumatic brain injury [44]. Although observational studies based on first postoperative sampling time showed no significant correlation between NSE and POCD, the pooled data showed that NSE was significantly correlated with POCD at 1 hour, 6 hours, 24 hours, 2 days, and 9 days postoperatively. The inconsistency of the results may be due to the different sampling times of the markers. In the included studies, Linstedt et al. [28] reported that the first sampling time of markers was 30 minutes postoperatively, while other studies were at 1 hour to 2 days postoperatively. Sensitivity analysis showed that when the study by Linstedt et al. [28] was removed, NSE was significantly correlated with POCD (*P*<0.0001).

Aβ is derived from the proteolysis of the β-amyloid precursor protein [45]. Progressive accumulation of Aβ is a pathological feature of AD. The accumulation of Aβ in synaptic mitochondria can damage neuronal function and is related to neuronal degeneration [45, 46]. In the present analysis, all of the studies involving the Aβ biomarker were observational studies, and the results showed that Aβ was not significantly associated with POCD according to the earliest postoperative sampling time-point. However, the pooled data showed that Aβ levels were significantly correlated with POCD at 24 hours, 2 days, and 9 days postoperatively. This may be due to the fact that the first sampling time of markers reported by Evered et al. [31] was 7 days postoperatively, which was significantly longer than that reported by other studies that sampled Aβ from 1 hour to 2 days postoperatively. In addition, the biomarkers used in this study were derived from the CSF, while the biomarkers of other studies were derived from blood. In particular, the Aβ value in this study was lower in the POCD group than in the non-POCD group, which differed from the conclusions of other studies that the biomarkers in the POCD group were higher than in the non-POCD group. The sensitivity analysis also showed that when the study by Evered et al. [31] was removed, Aβ was significantly correlated with POCD (*P* <0.00001).

Tau is a microtubule-associated protein in the brain and spinal cord that can stabilize axon microtubules [14]. In neurodegenerative diseases, such as AD, tau protein phosphorylation is related to nerve death [47]. Among the included studies, only one detected this biomarker; therefore, a pooled analysis of tau could not be performed. However, according to the results of Evered et al. [31] and a previous finding [21], tau is not significantly associated with POCD.

Neurofilaments are abundant structural scaffolding proteins that are expressed exclusively in neurons. Neurofilaments have attracted increasing attention as candidate biomarkers of axonal injury. NFL, a neurofilament, is considered the most promising biomarker for neurological diseases [48]. Similarly, only Evered et al. [31] detected this biomarker in the included studies. According to their results [21] and those of others [49], NFL has a limited predictive value for POCD.

GFAP is an important component of the astrocyte cytoskeleton and is associated with cell regeneration, synaptic plasticity, and reactive glial cell proliferation [50]. However, none of the included studies detected this marker. But, previous studies [21, 43, 51, 52] have shown that GFAP was not significantly associated with POCD.

In particular, two studies have different conclusions about whether the ratio of Aβ and tau is associated with postoperative neurocognitive decline. Berger et al. [53] showed that neurocognitive decline after noncardiac and non-neurosurgical operations is unlikely to be associated with changes in tau/Aβ ratios. However, Wu et al. [54] showed that the Aβ-42/tau ratio can be used to predict the development of POCD in the elderly. Therefore, further studies are required to address this issue.

In 2018, relevant experts presented recommendations [55] for the nomenclature of cognitive changes associated with anesthesia and surgery. Before renaming, changes in postoperative cognitive function were divided into delirium during the recovery period, postoperative delirium, and POCD. Similar to POCD, the relationship between postoperative delirium and neuronal injury biomarkers remains unclear. Ballweg et al. [56] have shown that changes in plasma tau protein are associated with the incidence and severity of delirium. Three studies [57–59] found that patients with elevated NFL levels were more prone to delirium. However, two studies [60, 61] found that postoperative delirium was not associated with Aβ1–42, tau, and S100β levels. According to the recommendations [55], using the term “neurocognitive disorders” to describe changes in cognitive function from the preoperative period to 12 months postoperatively will help reduce the impact caused by the different evaluation times of cognitive function.

To our knowledge, this is the first meta-analysis to explore the predictive value of neuronal injury biomarkers for POCD. The results of our analysis showed that the three biomarkers, S100β, NSE, and Aβ, have predictive value for POCD. The relationship between biomarkers and postoperative cognitive outcomes is closely related to their detection time.

This meta-analysis had some limitations. Firstly, heterogeneity was high in many analyses. Heterogeneity may be due to the following reasons: (1) small sample size of each study, (2) different sources (blood or CSF), detection time, and biomarker identification methods, (3) different evaluation times and diagnostic methods of POCD, (4) different types of surgery, and (5) patients of different ages. Secondly, the number of articles included was relatively small and most of the analyses were based on a few studies, which hindered us from conducting a more in-depth analysis of patient age, surgical type, anesthesia method, and source of marker samples. Thirdly, due to the lack of available data, we could not further analyze the impact of preoperative biomarker levels on the present findings. Fourthly, due to the inconsistent diagnosis time of POCD, the predictive value of biomarkers for postoperative short- and long-term cognitive function could not be compared. Therefore, future studies should focus on these issues.

Biomarkers derived from CSF are considered to be the gold standard for POCD-related biomarkers. However, due to the difficulty of extracting CSF samples from patients taking anticoagulant drugs or emergency patients, their clinical application is limited [15]. This means that blood samples may be an easier source than CSF. Among the studies included in this analysis, only one study [31] sample was derived from CSF, which may also be explained by the above reasons. Cata et al. [14] proposed that the S100β level at 24 hours after surgery has high sensitivity and specificity in identifying patients with brain injury; tau protein levels peaked at 6 hours after surgery and returned to baseline levels on the fourth day after surgery. The study by Herrmann et al. [62] showed that concentrations of NSE returned to baseline at 24 hours after surgery. However, our meta-analysis showed no significant correlation between S100β and POCD at 6 hours and 24 hours after surgery. Future studies are necessary to optimize the sampling time point for the better prediction of POCD. According to the recommendations by Evered et al. [55], the objective criteria of POCD should be based on one or more cognitive domains, including attention, executive function, learning and memory, language, perceptual movement, or social cognition. Compared to screening tools, such as the Mini-mental State Examination or Montreal Cognitive Assessment, using psychometric assessments can objectively assess specific cognitive domains. And the time to evaluate cognitive function should be within 30 days to 12 months after surgery. Future studies are warranted to unify the source of biomarkers, sampling time points, and diagnostic methods of POCD so as to further demonstrate the predictive value of biomarkers in perioperative cognitive function.

Early identification of patients with high risk of POCD helps facilitate preventive measures to reduce the incidence of disease, accelerate postoperative rehabilitation, shorten hospitalization time, and reduce medical costs. Early enteral feeding, postoperative multimodal analgesia, increased physical activity, and social participation, as well as care for patients in a quiet environment with the presence of family members, can help improve the cognitive function of the elderly. However, the therapeutic effect of related drugs on POCD, including memantine, huperzine A, and brain-derived neurotrophic factor, remains to be further confirmed [1].

This meta-analysis identified that postoperative S100β, NSE, and Aβ levels have predictive value for POCD. The relationship between these biomarkers and POCD may be affected by sampling time. Early diagnosis and prevention of POCD are important in postoperative clinical practice, and future studies with larger sample sizes are warranted to confirm this finding.

## Supporting information

S1 Appendix PRISMA checklist. (DOC)

S2 Appendix The data used for analyses. (XLSX)

S3 Appendix The Newcastle-Ottawa Quality Assessment Scale was used to evaluate the quality of the observational study. (DOCX)

S4 Appendix Risk of bias assessment for the individual randomized controlled trial according to the Cochrane collaboration tool. (DOCX)

S5 Appendix Egger’s test results for publication and selective reporting bias. (DOCX)

## References

[1] Kotekar N, Shenkar A, Nagaraj R. Postoperative cognitive dysfunction–current preventive strategies. Clin Interv Aging. 2018;13: 2267–2273. doi: 10.2147/CIA.S133896 30519008 PMC6233864

[2] Androsova G, Krause R, Winterer G, Schneider R. Biomarkers of postoperative delirium and cognitive dysfunction. Front Aging Neurosci. 2015;7: 112. doi: 10.3389/fnagi.2015.00112 26106326 PMC4460425

[3] Lin X, Chen Y, Zhang P, Chen G, Zhou Y, Yu X. The potential mechanism of postoperative cognitive dysfunction in older people. Exp Gerontol. 2020;130: 110791. doi: 10.1016/j.exger.2019.110791 31765741

[4] Evered L, Atkins K, Silbert B, Scott DA. Acute peri-operative neurocognitive disorders: a narrative review. Anaesthesia. 2022;77(suppl 1): 34–42. doi: 10.1111/anae.15613 35001385

[5] Borchers F, Spies CD, Feinkohl I, Brockhaus WR, Kraft A, Kozma P, et al. Methodology of measuring postoperative cognitive dysfunction: a systematic review. Br J Anaesth. 2021;126: 1119–1127. doi: 10.1016/j.bja.2021.01.035 33820655

[6] Liu J, Huang K, Zhu B, Zhou B, Ahmad Harb AK, Liu L, et al. Neuropsychological tests in post-operative cognitive dysfunction: methods and applications. Front Psychol. 2021;12: 684307. doi: 10.3389/fpsyg.2021.684307 34149572 PMC8212929

[7] Reinsfelt B, Westerlind A, Blennow K, Zetterberg H, Ricksten SE. Open-heart surgery increases cerebrospinal fluid levels of Alzheimer-associated amyloid β. Acta Anaesthesiol Scand. 2013;57: 82–88. doi: 10.1111/j.1399-6576.2012.02769.x 22998015

[8] Rappold T, Laflam A, Hori D, Brown C, Brandt J, Mintz CD, et al. Evidence of an association between brain cellular injury and cognitive decline after non-cardiac surgery. Br J Anaesth. 2016;116: 83–89. doi: 10.1093/bja/aev415 26675953 PMC4681618

[9] McKhann GM, Knopman DS, Chertkow H, Hyman BT, Jack CR, Kawas CH, et al. The diagnosis of dementia due to Alzheimer’s disease: recommendations from the National Institute on Aging-Alzheimer’s Association workgroups on diagnostic guidelines for Alzheimer’s disease. Alzheimers Dement. 2011;7: 263–269. doi: 10.1016/j.jalz.2011.03.005 21514250 PMC3312024

[10] Osborn KE, Khan OA, Kresge HA, Bown CW, Liu D, Moore EE, et al. Cerebrospinal fluid and plasma neurofilament light relate to abnormal cognition. Alzheimers Dement (Amst). 2019;11: 700–709. doi: 10.1016/j.dadm.2019.08.008 31700989 PMC6827361

[11] Gaiottino J, Norgren N, Dobson R, Topping J, Nissim A, Malaspina A, et al. Increased neurofilament light chain blood levels in neurodegenerative neurological diseases. PLOS ONE. 2013;8: e75091. doi: 10.1371/journal.pone.0075091 24073237 PMC3779219

[12] Gaetani L, Blennow K, Calabresi P, Di Filippo M, Parnetti L, Zetterberg H. Neurofilament light chain as a biomarker in neurological disorders. J Neurol Neurosurg Psychiatry. 2019;90: 870–881. doi: 10.1136/jnnp-2018-320106 30967444

[13] Narayanan S, Shanker A, Khera T, Subramaniam B. Neurofilament light: a narrative review on biomarker utility. FAC Rev. 2021;10: 46. doi: 10.12703/r/10-46 34131656 PMC8170685

[14] Cata JP, Abdelmalak B, Farag E. Neurological biomarkers in the perioperative period. Br J Anaesth. 2011;107: 844–858. doi: 10.1093/bja/aer338 22065690

[15] Schaefer ST, Koenigsperger S, Olotu C, Saller T. Biomarkers and postoperative cognitive function: could it be that easy? Curr Opin Anaesthesiol. 2019;32: 92–100. doi: 10.1097/ACO.0000000000000676 30507679

[16] Tomaszewski D. Biomarkers of brain damage and postoperative cognitive disorders in orthopedic patients: an update. BioMed Res Int. 2015;2015: 402959. doi: 10.1155/2015/402959 26417595 PMC4568345

[17] Sun Y, Qin Q, Shang YJ, Fang CP, Zhang WW, Gu ML, et al. The accuracy of glial fibrillary acidic protein in acute stroke differential diagnosis: A meta-analysis. Scand J Clin Lab Investig. 2013;73: 601–606. doi: 10.3109/00365513.2013.830326 24200345

[18] Schiff L, Hadker N, Weiser S, Rausch C. A literature review of the feasibility of glial fibrillary acidic protein as a biomarker for stroke and traumatic brain injury. Mol Diagn Ther. 2012;16: 79–92. doi: 10.2165/11631580-000000000-00000 22497529

[19] Hall RJ, Watne LO, Cunningham E, Zetterberg H, Shenkin SD, Wyller TB, et al. CSF biomarkers in delirium: a systematic review. Int J Geriatr Psychiatry. 2018;33: 1479–1500. doi: 10.1002/gps.4720 28585290

[20] Danielson M, Wiklund A, Granath F, Blennow K, Mkrtchian S, Nellgård B, et al. Association between cerebrospinal fluid biomarkers of neuronal injury or amyloidosis and cognitive decline after major surgery. Br J Anaesth. 2021;126: 467–476. doi: 10.1016/j.bja.2020.09.043 33183737

[21] Wiberg S, Holmgaard F, Zetterberg H, Nilsson JC, Kjaergaard J, Wanscher M, et al. Biomarkers of cerebral injury for prediction of postoperative cognitive dysfunction in patients undergoing cardiac surgery. J Cardiothorac Vasc Anesth. 2022;36: 125–132. doi: 10.1053/j.jvca.2021.05.016 34130895

[22] Barbu M, Jónsson K, Zetterberg H, Blennow K, Kolsrud O, Ricksten SE, et al. Serum biomarkers of brain injury after uncomplicated cardiac surgery: secondary analysis from a randomized trial. Acta Anaesthesiol Scand. 2022;66: 447–453. doi: 10.1111/aas.14033 35118644 PMC9302991

[23] Wan X, Wang W, Liu J, Tong T. Estimating the sample mean and standard deviation from the sample size, median, range and/or interquartile range. BMC Med Res Methodol. 2014;14: 135. doi: 10.1186/1471-2288-14-135 25524443 PMC4383202

[24] Higgins JP, White IR, Anzures-Cabrera J. Meta-analysis of skewed data: combining results reported on log-transformed or raw scales. Stat Med. 2008;27: 6072–6092. doi: 10.1002/sim.3427 18800342 PMC2978323

[25] Hozo SP, Djulbegovic B, Hozo I. Estimating the mean and variance from the median, range, and the size of a sample. BMC Med Res Methodol. 2005;5: 13. doi: 10.1186/1471-2288-5-13 15840177 PMC1097734

[26] Sterne JAC, Savović J, Page MJ, Elbers RG, Blencowe NS, Boutron I, et al. RoB 2: a revised tool for assessing risk of bias in randomised trials. BMJ. 2019;366: l4898. doi: 10.1136/bmj.l4898 31462531

[27] Wells G, Shea B, O’Connell D, Peterson J, Welch V, Losos M, et al. The Newcastle-Ottawa Scale (NOS) for assessing the quality of nonrandomised studies in meta-analyses. Appl Eng Agric. 2014;18: 727–734.

[28] Linstedt U, Meyer O, Kropp P, Berkau A, Tapp E, Zenz M. Serum concentration of S-100 protein in assessment of cognitive dysfunction after general anesthesia in different types of surgery. Acta Anaesthesiol Scand. 2002;46: 384–389. doi: 10.1034/j.1399-6576.2002.460409.x 11952437

[29] Li YC, Xi CH, An YF, Dong WH, Zhou M. Perioperative inflammatory response and protein S-100β concentrations–relationship with post-operative cognitive dysfunction in elderly patients. Acta Anaesthesiol Scand. 2012;56: 595–600. doi: 10.1111/j.1399-6576.2011.02616.x 22224444

[30] Li XM, Shao MT, Wang JJ, Wang YL. Relationship between post-operative cognitive dysfunction and regional cerebral oxygen saturation and β-amyloid protein. J Zhejiang Univ Sci B. 2014;15: 870–878. doi: 10.1631/jzus.B1400130 25294376 PMC4201315

[31] Evered L, Silbert B, Scott DA, Ames D, Maruff P, Blennow K. Cerebrospinal fluid biomarker for Alzheimer disease predicts postoperative cognitive dysfunction. Anesthesiology. 2016;124: 353–361. doi: 10.1097/ALN.0000000000000953 26580833

[32] Yu P, Wang H, Mu L, Ding X, Ding W. Effect of general anesthesia on serum β-amyloid protein and regional cerebral oxygen saturation of elderly patients after subtotal gastrectomy. Exp Ther Med. 2016;12: 3561–3566. doi: 10.3892/etm.2016.3814 28101151 PMC5228211

[33] Cao YH, Chi P, Zhao YX, Dong XC. Effect of bispectral index-guided anesthesia on consumption of anesthetics and early postoperative cognitive dysfunction after liver transplantation: an observational study. Medicine. 2017;96: e7966. doi: 10.1097/MD.0000000000007966 28858130 PMC5585524

[34] Chi YL, Li ZS, Lin CS, Wang Q, Zhou YK. Evaluation of the postoperative cognitive dysfunction in elderly patients with general anesthesia. Eur Rev Med Pharmacol Sci. 2017;21: 1346–1354. 28387891

[35] He X, Wen LJ, Cui C, Li DR, Teng JF. The significance of S100β protein on postoperative cognitive dysfunction in patients who underwent single valve replacement surgery under general anesthesia. Eur Rev Med Pharmacol Sci. 2017;21: 2192–2198. 28537663

[36] Kok WF, Koerts J, Tucha O, Scheeren TWL, Absalom AR. Neuronal damage biomarkers in the identification of patients at risk of long-term postoperative cognitive dysfunction after cardiac surgery. Anaesthesia. 2017;72: 359–369. doi: 10.1111/anae.13712 27987229

[37] Baktiar Y, Soenarto RF, Alatas A, Auerkari AN. S100B as a serologic marker for cognitive dysfunction following open-heart surgery. Int J Appl Pharm. 2020: 50–53. doi: 10.22159/ijap.2020.v12s3.39473 Ballweg. v12s3.39473: 50–53; 2020.

[38] Wan Z, Li Y, Ye H, Zi Y, Zhang G, Wang X. Plasma S100β and neuron-specific enolase, but not neuroglobin, are associated with early cognitive dysfunction after total arch replacement surgery: a pilot study Medicine. 2021;100: e25446. doi: 10.1097/MD.0000000000025446 33847649 PMC8051968

[39] Ntalouka MP, Arnaoutoglou E, Tzimas P. Postoperative cognitive disorders: an update. Hippokratia. 2018;22: 147–154. 31695301 PMC6825421

[40] Moore BW. A soluble protein characteristic of the nervous system. Biochem Biophys Res Commun. 1965;19: 739–744. doi: 10.1016/0006-291x(65)90320-7 4953930

[41] Silva FP, Schmidt AP, Valentin LS, Pinto KO, Zeferino SP, Oses JP, et al. S100B protein and neuron-specific enolase as predictors of cognitive dysfunction after coronary artery bypass graft surgery: A prospective observational study. Eur J Anaesthesiol. 2016;33: 681–689. doi: 10.1097/EJA.0000000000000450 27433840

[42] Goettel N, Burkhart CS, Rossi A, Cabella BC, Berres M, Monsch AU, et al. Associations between impaired cerebral blood flow autoregulation, cerebral oxygenation, and biomarkers of brain injury and postoperative cognitive dysfunction in elderly patients after major noncardiac surgery. Anesth Analg. 2017;124: 934–942. doi: 10.1213/ANE.0000000000001803 28151820

[43] Danielson M, Wiklund A, Granath F, Blennow K, Mkrtchian S, Nellgård B, et al. Neuroinflammatory markers associate with cognitive decline after major surgery: findings of an explorative study. Ann Neurol. 2020;87: 370–382. doi: 10.1002/ana.25678 31930549

[44] Isgrò MA, Bottoni P, Scatena R. Neuron-specific enolase as a biomarker: biochemical and clinical aspects. Adv Exp Med Biol. 2015;867: 125–143. doi: 10.1007/978-94-017-7215-0_9 26530364

[45] Kumar S, Wirths O, Theil S, Gerth J, Bayer TA, Walter J. Early intraneuronal accumulation and increased aggregation of phosphorylated Abeta in a mouse model of Alzheimer’s disease. Acta Neuropathol. 2013;125: 699–709. doi: 10.1007/s00401-013-1107-8 23525537

[46] Yan SF, Akhter F, Sosunov AA, Yan SS. Identification and characterization of amyloid-beta accumulation in synaptic mitochondria. Methods Mol Biol. 2018;1779: 415–433. doi: 10.1007/978-1-4939-7816-8_25 29886547

[47] Hanger DP, Seereeram A, Noble W. Mediators of tau phosphorylation in the pathogenesis of Alzheimer’s disease. Expert Rev Neurother. 2009;9: 1647–1666. doi: 10.1586/ern.09.104 19903024

[48] Khalil M, Teunissen CE, Otto M, Piehl F, Sormani MP, Gattringer T, et al. Neurofilaments as biomarkers in neurological disorders. Nat Rev Neurol. 2018;14: 577–589. doi: 10.1038/s41582-018-0058-z 30171200

[49] Larsen JR, Kobborg T, Shahim P, Blennow K, Rasmussen LS, Zetterberg H. Serum-neuroproteins, near-infrared spectroscopy, and cognitive outcome after beach-chair shoulder surgery: observational cohort study analyses. Acta Anaesthesiol Scand. 2021;65: 26–33. doi: 10.1111/aas.13691 32812646

[50] Middeldorp J, Hol EM. GFAP in health and disease. Prog Neurobiol. 2011;93: 421–443. doi: 10.1016/j.pneurobio.2011.01.005 21219963

[51] Kumpaitiene B, Svagzdiene M, Drigotiene I, Sirvinskas E, Sepetiene R, Zakelis R, et al. Correlation among decreased regional cerebral oxygen saturation, blood levels of brain injury biomarkers, and cognitive disorder. J Int Med Res. 2018;46: 3621–3629. doi: 10.1177/0300060518776545 29896989 PMC6136023

[52] Szwed K, Słomka A, Pawliszak W, Szwed M, Anisimowicz L, Żekanowska E, et al. Novel markers for predicting Type 2 neurologic complications of coronary artery bypass grafting. Ann Thorac Surg. 2020;110: 599–607. doi: 10.1016/j.athoracsur.2019.10.071 31863758

[53] Berger M, Browndyke JN, Cooter Wright M, Nobuhara C, Reese M, Acker L, et al. Postoperative changes in cognition and cerebrospinal fluid neurodegenerative disease biomarkers. Ann Clin Transl Neurol. 2022;9: 155–170. doi: 10.1002/acn3.51499 35104057 PMC8862419

[54] Wu Z, Zhang M, Zhang Z, Dong W, Wang Q, Ren J. Ratio of β-amyloid protein (Aβ) and Tau predicts the postoperative cognitive dysfunction on patients undergoing total hip/knee replacement surgery. Exp Ther Med. 2018;15: 878–884. doi: 10.3892/etm.2017.5480 29399093 PMC5772777

[55] Evered L, Silbert B, Knopman DS, Scott DA, DeKosky ST, Rasmussen LS, et al. Recommendations for the nomenclature of cognitive change associated with anaesthesia and surgery-2018. Br J Anaesth. 2018;121: 1005–1012. doi: 10.1016/j.bja.2017.11.087 30336844 PMC7069032

[56] Ballweg T, White M, Parker M, Casey C, Bo A, Farahbakhsh Z, et al. Association between plasma tau and postoperative delirium incidence and severity: a prospective observational study. Br J Anaesth. 2021;126: 458–466. doi: 10.1016/j.bja.2020.08.061 33228978 PMC8014913

[57] Halaas NB, Blennow K, Idland AV, Wyller TB, Ræder J, Frihagen F, et al. Neurofilament light in serum and cerebrospinal fluid of hip fracture patients with delirium. Dement Geriatr Cogn Disord. 2018;46: 346–357. doi: 10.1159/000494754 30522125

[58] Casey CP, Lindroth H, Mohanty R, Farahbakhsh Z, Ballweg T, Twadell S, et al. Postoperative delirium is associated with increased plasma neurofilament light. Brain. 2020;143: 47–54. doi: 10.1093/brain/awz354 31802104 PMC6935744

[59] Fong TG, Vasunilashorn SM, Ngo L, Libermann TA, Dillon ST, Schmitt EM, et al. Association of plasma neurofilament light with postoperative delirium. Ann Neurol. 2020;88: 984–994. doi: 10.1002/ana.25889 32881052 PMC7581557

[60] Witlox J, Kalisvaart KJ, de Jonghe JFM, Verwey NA, van Stijn MFM, Houdijk APJ, et al. Cerebrospinal fluid β-amyloid and tau are not associated with risk of delirium: a prospective cohort study in older adults with hip fracture. J Am Geriatr Soc. 2011;59: 1260–1267. doi: 10.1111/j.1532-5415.2011.03482.x 21718268

[61] Beishuizen SJ, Scholtens RM, van Munster BC, de Rooij SE. Unraveling the relationship between delirium, brain damage, and subsequent cognitive decline in a cohort of individuals undergoing surgery for hip fracture. J Am Geriatr Soc. 2017;65: 130–136. doi: 10.1111/jgs.14470 27641367

[62] Herrmann M, Ebert AD, Galazky I, Wunderlich MT, Kunz WS, Huth C. Neurobehavioral outcome prediction after cardiac surgery: role of neurobiochemical markers of damage to neuronal and glial brain tissue. Stroke. 2000;31: 645–650. doi: 10.1161/01.str.31.3.645 10700498

