# Supplementary material for: Relationship between postoperative biomarkers of neuronal injury and postoperative cognitive dysfunction: A meta-analysis
Source: PLoS One. 2023 Apr 25;18(4):e0284728. doi: 10.1371/journal.pone.0284728 (PMC10128950; doi:10.1371/journal.pone.0284728)
Supplement: S3 Appendix — (DOCX) [file pone.0284728.s003.docx]

**Appendix 3** The Newcastle-Ottawa Quality Assessment Scale was used to evaluate the quality of the observational study

|  | Selection | | | | Comparability | | Exposure | | |  |
| --- | --- | --- | --- | --- | --- | --- | --- | --- | --- | --- |
|  | #1 | #2 | #3 | #4 | #5 | #6 | #7 | #8 | #9 | Total |
| Ying-Hao Cao 2017 | ☆ | ☆ | ☆ | ☆ |  |  | ☆ | ☆ | ☆ | 7 |
| PENG YU 2016 | ☆ | ☆ | ☆ | ☆ | ☆ |  | ☆ | ☆ | ☆ | 8 |
| Y-C. Li 2012 | ☆ | ☆ | ☆ | ☆ |  |  | ☆ | ☆ |  | 6 |
| Xi-ming LI 2014 | ☆ | ☆ | ☆ | ☆ | ☆ | ☆ | ☆ | ☆ | ☆ | 9 |
| X. HE 2017 | ☆ | ☆ | ☆ | ☆ |  |  | ☆ | ☆ | ☆ | 7 |
| Zilin Wan 2021 | ☆ | ☆ | ☆ | ☆ | ☆ | ☆ | ☆ | ☆ | ☆ | 9 |
| YOSHUA BAKTIAR 2020 | ☆ | ☆ | ☆ | ☆ | ☆ |  | ☆ | ☆ | ☆ | 8 |
| U. LINSTEDT 2002 | ☆ | ☆ | ☆ | ☆ |  |  | ☆ | ☆ | ☆ | 7 |
| Lisbeth Evered 2016 | ☆ | ☆ | ☆ | ☆ |  |  | ☆ | ☆ | ☆ | 7 |
| W. F. Kok 2017 | ☆ | ☆ | ☆ | ☆ | ☆ | ☆ | ☆ | ☆ |  | 8 |

#1 Diagnosis of POCD

#2 Representativeness of patients with POCD

#3 Selection of the Comparative patients without non-POCD

#4 Definition of Controls

#5 Study controls for age

#6 Study controls for Gender/Type of surgery/Type of anesthesia/Education years

#7 Ascertainment of POCD station

#8 Same method of ascertainment for POCD station

#9 Lost to follow up acceptable
