# Supplementary material for: Relationship between postoperative biomarkers of neuronal injury and postoperative cognitive dysfunction: A meta-analysis
Source: PLoS One. 2023 Apr 25;18(4):e0284728. doi: 10.1371/journal.pone.0284728 (PMC10128950; doi:10.1371/journal.pone.0284728)

**Appendix 4** Risk of bias assessment for the individual randomized controlled trial according to the Cochrane collaboration tool


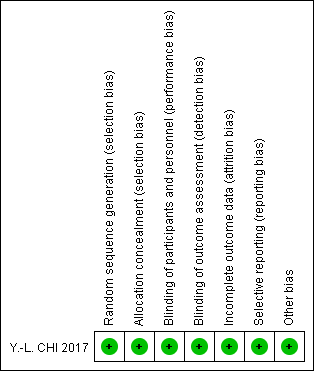

Supplement: S4 Appendix — (DOCX) [file pone.0284728.s004.docx]
