# Supplementary material for: Relationship between postoperative biomarkers of neuronal injury and postoperative cognitive dysfunction: A meta-analysis
Source: PLoS One. 2023 Apr 25;18(4):e0284728. doi: 10.1371/journal.pone.0284728 (PMC10128950; doi:10.1371/journal.pone.0284728)
Supplement: S5 Appendix — (DOCX) [file pone.0284728.s005.docx]

**Appendix 5** Egger’s test results for publication and selective reporting bias

| Egger’s test (bias) | | | | |
| --- | --- | --- | --- | --- |
| biomarker | Coef | 95%CI | t | p |
| S-100β | 2.34 | -3.16，7.83 | 1.09 | 0.324 |
| NSE | -3.01 | -11.98，5.96 | -1.44 | 0.286 |
| Aβ | 2.73 | -83.04，88.49 | 0.4 | 0.755 |
